# Measures of Agreement with Multiple Raters: Fréchet Variances and Inference

**DOI:** 10.1007/s11336-023-09945-2

**Published:** 2024-01-08

**Authors:** Jonas Moss

**Affiliations:** Department of Data Science and Analytics, BI Norwegian Business School, Oslo, Norway

**Keywords:** agreement, inter-rater reliability, AC1, Cohen kappa

## Abstract

Most measures of agreement are chance-corrected. They differ in three dimensions: their definition of chance agreement, their choice of disagreement function, and how they handle multiple raters. Chance agreement is usually defined in a pairwise manner, following either Cohen’s kappa or Fleiss’s kappa. The disagreement function is usually a nominal, quadratic, or absolute value function. But how to handle multiple raters is contentious, with the main contenders being Fleiss’s kappa, Conger’s kappa, and Hubert’s kappa, the variant of Fleiss’s kappa where agreement is said to occur only if every rater agrees. More generally, multi-rater agreement coefficients can be defined in a *g*-wise way, where the disagreement weighting function uses *g* raters instead of two. This paper contains two main contributions. (a) We propose using Fréchet variances to handle the case of multiple raters. The Fréchet variances are intuitive disagreement measures and turn out to generalize the nominal, quadratic, and absolute value functions to the case of more than two raters. (b) We derive the limit theory of *g*-wise weighted agreement coefficients, with chance agreement of the Cohen-type or Fleiss-type, for the case where every item is rated by the same number of raters. Trying out three confidence interval constructions, we end up recommending calculating confidence intervals using the arcsine transform or the Fisher transform.

**Supplementary Information:**

The online version contains supplementary material available at 10.1007/s11336-023-09945-2.

## Introduction

The most popular measures of inter-rater agreement involve correction for chance agreement. These can be written on the form

1.1 $$\begin{aligned} \frac{p_{a}-p_{ca}}{1-p_{ca}}=1-\frac{p_{d}}{p_{cd}}, \end{aligned}$$

where $p_{a}$ ($p_{d}$) is the percentage agreement (disagreement) between the raters and $p_{ca}$ ($p_{cd}$) is the chance agreement (disagreement) between the raters. Such measures are frequently called *chance-corrected measures of agreement*. Well-known examples of coefficients in this class are Cohen’s (1960) kappa and its weighted variant (1968), its multi-rater variant Conger’s kappa (Conger, 1980; Light, 1971), Krippendorff’s (1970) alpha, Scott’s (1955) pi, and Fleiss’ (1971) kappa. Some of these coefficients are defined only for two raters. The rest are defined in a pairwise manner, in the sense that they measure agreement between two raters at a time. However, not every proposed measure of agreement is defined on pairs of raters. The most famous is Hubert’s kappa (1977), which was recently studied in detail by Martín Andrés and Álvarez Hernández (2020). Other agreement coefficients include the $AC_{1}$ coefficient (Gwet, 2008), the recent coefficient of van Oest (2019), and a multitude of intraclass correlation coefficients (Gwet, 2014).

There is no consensus on how multi-rater agreement coefficients should be defined. Broadly speaking, two options are considered: pairwise coefficients and consensus coefficients. The pairwise coefficients measure the agreement between pairs of raters (Conger, 1980), while the consensus coefficients measure the simultaneous agreement between all raters. In particular, consensus coefficients support the notion that “agreement occurs if and only if all raters agree on the categorization of an object” (Hubert, 1977). Both pairwise and consensus-based definitions of agreement are variants of *g*-wise measures of agreement (Conger, 1980), where agreement is measured among *g*-tuples of raters. The case where $2<g<R$ has received little attention in the literature (Warrens, 2012), and non-trivial ways to measure agreement are hard to invent in this case. However, we introduce a promising and general framework for handling *g*-wise measures of agreement based on the concept of *Fréchet variances* (Dubey and Müller, 2019). The Fréchet variances generalize the variance and the measures of agreement based on them generalize the nominal, linearly weighted, and quadratically weighted pairwise measures of agreement in a natural way. They are easily interpretable, as you measure how much the raters disagree with the generalized mean rater and then adjust for chance. For nominal data in particular, they measure how many raters disagree with the modal rater, with a resulting agreement measure less extreme than Hubert’s kappa.

We need inferential theory for the *g*-wise agreement coefficients to make them useful. Much work has been done on inference for agreement coefficients, but, to our knowledge, inference for *g*-wise agreement coefficients has yet to be studied. Assuming multivariate normality of the ratings, Lin (1989, Section 3) derived the asymptotic distribution of Cohen’s kappa with quadratic weights. Fleiss (1971) introduced a formula for the standard error of Fleiss’s kappa, but later showed that it was incorrect. Using the properties of the multinomial distribution and the delta method, Schouten (1980) found the asymptotic variance of the weighted Fleiss’s kappa in the case when the number of categories is finite. Almost forty years later, Gwet (2021) found a consistent estimator of the variance for the unweighted Fleiss’s kappa. We extend these results to the weighted *g*-wise Fleiss’s kappa for any number of categories below. In addition, we mention that bootstrap inference for Fleiss’s kappa and Krippendorff’s alpha was studied by Zapf et al. (2016).

We begin the paper by providing the definitions of two kinds of chance-corrected agreement coefficients. Then, in Sect. 2, we establish connections between the multi-rater Cohen’s kappa, Fleiss’s kappa, Conger’s kappa, Krippendorff’s alpha, and Hubert’s kappa. We restrict ourselves to the context where every rater rates every item. In Sect. 3, we discuss the Fréchet variances mentioned above. Then we spell out the basic limit theory for this class agreement coefficients in Sect. 4, extending the results of Schouten (1980), Schouten (1982), and O’Connell and Dobson (1984) to vector-valued items and *g*-wise coefficients. We do this using the theory of *U*-statistics (Lee, 2019), but there are other ways to arrive at the same results. Then, in Sect. 5, we provide practical recommendations regarding the choice of confidence interval, obtained by comparing three confidence interval constructions: basic, arcsine transformed, and Fisher transformed. Using a simulation study, we find that the arcsine and Fisher intervals outperform the basic interval when *n* is small.

## Measures of Agreement

Let $d(x_{1},\ldots ,x_{g})$ be a *disagreement function*, a positive and symmetric function of *g* arguments that equals 0 when all $x_{i}$s are equal, i.e., $d(x,\ldots ,x)=0$. The disagreement function quantifies the disagreement between the ratings $x_{1},\ldots ,x_{g}$, where 0 is understood as complete agreement.

Most disagreement functions take two arguments. While there are infinitely many disagreement functions, the best-known belong to the class of $l_{p}$ quasi-norms, $p=0,1,2$, potentially raised to the *p*th power. The $l_{p}$ quasi-norms, $p\in [0,\infty ]$ in $\mathbb {R}^{k}$ are defined as

2.1 $$\begin{aligned} \Vert x\Vert _{p}=\left( \sum _{i=1}^{k}|x_{i}|^{p}\right) ^{1/p}. \end{aligned}$$

Here $||x||_{0}=\sum _{i=1}^{k}1[x_{i}\ne 0]$ and $||x||_{\infty }=\sup _i |x_{i}|$, as can be verified by taking the limit of $||x||_{p}$ as $p\rightarrow 0$ and $p\rightarrow \infty$, respectively. It is well known that $||x||_{p}$ are proper norms if and only if $p\ge 1$, as the triangle inequality is violated when $1>p\ge 0$.

Now define the disagreement functions $d_{p}$ as the $l_{p}$ quasi-norm evaluated in $x_{1}-x_{2}$, i.e.,

2.2 $$\begin{aligned} d_{p}(x_{1},x_{2})=||x_{1}-x_{2}||_{p}. \end{aligned}$$

In the case of scalar values, $d_{0}(x_{1},x_{2})=1[x_{1}\ne x_{2}]$ is known as the *nominal disagreement function*. For $p=1$, the $l_{p}$ norm equals $d_{1}(x_{1},x_{2})=|x_{1}-x_{2}|$, which is known as the *absolute value disagreement function* (and sometimes the linear disagreement function). The *quadratic disagreement function* is $d_{2}^{2}(x_{1},x_{2})=(x_{1}-x_{2})^{2}$. Vector-valued variants of $d_{p}$ and $d_{p}^{p}$ are much less common, but have been used by, e.g., Berry et al. (2008).

When the dimension of the disagreement function *d* is not equal to 2, we are mostly interested in the case where its dimension equals the number of raters *R*. In this case, the disagreement functions often measure the degree of consensus among the raters, with 0 reflecting complete consensus. The most obvious choice is the *Hubert disagreement function*,

2.3 $$\begin{aligned} d(x_{1},\ldots ,x_{g})=1-1[x_{1}=\cdots =x_{g}] \end{aligned}$$

which equals 0 if and only if every rater agrees on a rating. The disagreement function is employed in Hubert’s kappa (Hubert, 1977).

We present our results in terms of disagreement functions instead of the more popular agreement functions (i.e., positive symmetric functions bounded by 1 where 1 signifies maximal agreement, sometimes with the additional assumption that $a\ge 0$). We do this mainly for mathematical convenience. Agreement functions and disagreement functions are closely related, for if *a* is an agreement function, then $d=1-a$ is a disagreement function. Our results could have been framed in terms of agreement functions instead, though with some loss of generality. See Appendix (Sect. 6) for a short discussion.

Our results and definitions are framed in the following setup. Let *R* be the number of raters and *n* be the number of items rated. Moreover, let *F* be a fixed multivariate distribution function *F* so that all rating vectors $\varvec{X}_{i}$ are sampled independently from *F*. In symbols,

2.4 $$\begin{aligned} \varvec{X}_{1},\varvec{X}_{2},\ldots ,\varvec{X}_{n} {\mathop {\sim }\limits ^{iid}} F. \end{aligned}$$

There are no restrictions on the rating vector components $\varvec{X}_{ir}$. They can be, e.g., categorical, real numbers, or vectors.

Equation (2.4) implies that every item is rated by exactly the same number of raters, which we refer to as the *rectangular design* assumption. The assumption is common in the literature,^1^ but far from universal. It can be relaxed, but it is strictly required for the limit results. We sketch how to loosen it in Appendix (Sect. 6), but we have made no attempts at an inferential theory for non-rectangular designs.

There are two important special cases covered by equation (2.4). First, in the case of *fixed raters*, the same set of ordered raters rate every item. Having fixed raters is common in applications of Cohen’s kappa, Conger’s kappa, and the concordance correlation coefficient.^2^ Having fixed raters ensures that *F* does not vary across different rating vectors, but *F* could potentially vary with the ratings when the raters are not fixed, provided we do not make further assumptions. And that leads us to the second case, that of *exchangeable ratings* given the item. Here, the rater identities do not affect the ratings given. The raters may be different for each item, but the distribution *F* will still be fixed. Exchangeable ratings occur when the ratings are identically distributed conditional on the item rated. Exchangeable ratings is an implicit assumption underlying most applications of Fleiss’ kappa, e.g., that of Fleiss (1971). In this case, the marginal distributions for all raters will be equal, which implies that the population value of the generalized Fleiss kappa equals the population value of the generalized Cohen’s kappa, both defined below. However, the sample Fleiss’s kappa is the preferred sample estimator, as it is invariant under changes of the raters’ identities.

We intend to collect the kappas of Cohen, Fleiss, Conger, Hubert, and so on, into a coherent framework of *g*-wise agreement coefficients. To do this, we will have to define some quantities. Let $\varvec{x}_{i}=(x_{i1},x_{i2},\ldots ,x_{iR})$ be an *R*-dimensional vector of observed ratings, and recall that *g* is the dimension of our disagreement function *d*. The following definitions are natural population counterparts of sample definitions prevalent in the agreement literature.

(i) **The disagreement at** $\varvec{x}_{1}$, as measured by *d*. The purpose of this quantity is to translate an arbitrary *g*-dimensional disagreement function *d* into a disagreement function taking an *R*-dimensional vector $\varvec{x}_{1}$ as input. It is defined as
  2.5 $$\begin{aligned} D_{d}(\varvec{x}_{1})=\left( {\begin{array}{c}R\\ g\end{array}}\right) ^{-1} \sum _{r_{1},\ldots ,r_{g}}d(\varvec{x}_{1r_{1}},\ldots , \varvec{x}_{1r_{g}}), \end{aligned}$$
  where the sum runs over all *g*-dimensional subsets of $\{1,\ldots ,R\}$ with order ignored, i.e., the *g*-combinations of *R*. The expression is simplified when $g=R$, as $D_{d}(\varvec{x}_{1})=d(\varvec{x}_{11},\ldots ,\varvec{x}_{1R})$ in this case. To gain some intuition about this quantity, suppose that $g=2$, that $x_{1},x_{2}$ are scalars, and consider the nominal disagreement function $d_{0}(x_{1},x_{2})=1[x_{1}\ne x_{2}]$. Then $D_{d}(\varvec{x}_{1})=2R^{-1}(R-1)^{-1}\sum _{r_{1}>r_{2}}1[x_{1r_{1}}\ne x_{1r_{2}}]$ is the percentage of times two distinct raters disagree on their rating.
(ii) **The Cohen-type chance disagreement** at $\varvec{x}_{1},\ldots ,\varvec{x}_{g}$, so called to differentiate it from the Fleiss-type chance disagreement. It is similar to the disagreement at $\varvec{x}_{1}$, but this time the raters do not necessarily rate the same item, as one rater rates the first item (from $\varvec{x}_{1}$) another rater rates the second item (from $\varvec{x}_{2}$), and so on. We do not allow a rater to rate the same item more than once in a pass: Hence, we need to choose *g* raters from a set of *R* raters, and the chance disagreement is
  2.6 $$\begin{aligned} C_{d}(\varvec{x}_{1},\ldots ,\varvec{x}_{g}) =\left( {\begin{array}{c}R\\ g\end{array}}\right) ^{-1}\sum _{r_{1},\ldots ,r_{g}}d(\varvec{x}_{1r_{1}}, \ldots ,\varvec{x}_{gr_{g}}), \end{aligned}$$
  where the sum runs over all *g*-dimensional subsets of $\{1,\ldots ,R\}$, i.e., the *g*-combinations of *R*. Observe that $D_{d}(\varvec{x})=C_{d}(\varvec{x},,\ldots ,\varvec{x})$. Since *d* is assumed to be symmetric, the expression is simplified to $d(\varvec{x}_{1r_{1}},\ldots ,\varvec{x}_{Rr_{R}})$ when $g=R$. When $g=2$, $C_{d}(\varvec{x}_{1},\varvec{x}_{2})=R^{-1}(R-1)^{-1} \sum _{r_{1}\ne r_{2}}d(\varvec{x}_{1r_{1}},\varvec{x}_{2r_{2}})$.
(iii) **The Fleiss-type chance disagreement** at $\varvec{x}_{1},\ldots ,\varvec{x}_{g}$ is similar, but allows the same rater to rate an item multiple times. Its definition is
  2.7 $$\begin{aligned} F_{d}(\varvec{x}_{1},\ldots ,\varvec{x}_{g}) =R^{-g}\sum _{r_{1},\ldots ,r_{g}}d(\varvec{x}_{1r_{1}}, \ldots ,\varvec{x}_{gr_{g}}), \end{aligned}$$
  where the sum runs over the product set $R^{g}$. The expression for $F_{d}(\varvec{x}_{1},\ldots ,\varvec{x}_{g})$ is not dramatically simplified when $g=R$. When $g=2$, $F_{d}(\varvec{x}_{1},\varvec{x}_{2})=R^{-2} \sum _{r_{1},r_{2}}d(\varvec{x}_{1r_{1}},\varvec{x}_{2r_{2}})$.

We will call the expected values of these quantities the *mean disagreement*, the *mean Cohen-type chance disagreement*, and the *mean Fleiss-type chance disagreement*. Slightly abusing notation, we denote them as

2.8 $$\begin{aligned} D_{d}=E[D_{d}(\varvec{X}_{1})],\quad C_{d}=E[C_{d}(\varvec{X}_{1},\ldots ,\varvec{X}_{g})],\quad F_{d}=E[F_{d}(\varvec{X}_{1},\ldots ,\varvec{X}_{g})], \end{aligned}$$

where $\varvec{X}_{1},\ldots ,\varvec{X}_{g}$ are independently sampled from the same distribution *F*. Discussions about the difference between $E[C_{d}(\varvec{X}_{1},\ldots ,\varvec{X}_{g})]$ and $E[F_{d}(\varvec{X}_{1},\ldots ,\varvec{X}_{g})]$, and why to prefer one over the other, are abundant in the literature, often in the context of the so-called paradox of kappa (Cicchetti and Feinstein, 1990).

### Definition 1

Let $X\sim F$ be a vector of *R* ratings and *d* be an agreement function with dimension *g*. Define the population values of the generalized Cohen’s kappa $(\kappa _{d})$ and Fleiss’s kappa $(\pi _{d})$ as

2.9 $$\begin{aligned} \kappa _{d}=1-\frac{D_{d}}{C_{d}},\quad \pi _{d}=1 -\frac{D_{d}}{F_{d}}. \end{aligned}$$

The generalized Fleiss’s kappa, denoted as $\pi _{d}$ since it generalizes of Scott’s pi (Scott, 1955), is a straightforward generalization of the Fleiss kappa (1971) to hold for $2< g\le R$. When $g=R$ and *d* is the nominal disagreement, it equals Hubert’s kappa. Likewise, the generalized Cohen’s kappa is an extension of weighted Conger’s kappa to hold for $2\le g\le R$. When $g=R$, it equals the Schuster–Smith coefficient (Schuster & Smith, 2005, eq. 1).^3^ It generalizes several other agreement coefficients as well. For instance, Berry and Mielke (1988) discussed what we call $\kappa _{d}$ for Euclidean weights between vector-valued ratings, while Janson and Olsson (2001) extended it to squared Euclidean and nominal weights. The relationship between most of the mentioned agreement coefficients is summarized in Table 1.

**Table 1:** Weighted agreement coefficients.

Coefficient	$R=2$	$R>2$	
		$g=2$	$g=R$
Cohen-type ($\kappa _{d}$)	Cohen’s kappa	Conger’s kappa^†^	Schuster–Smith
	Lin’s CC*	CC*	
Fleiss-type ($\pi _{d}$)	Scott’s $\pi ^{\dagger }$	Fleiss’ kappa^†^	Hubert’s kappa^†^
		Krippendorff’s alpha	

*Lin’s concordance coefficient and the concordance correlation coefficient (CC) is defined for quadratic weights only.

^†^Originally defined for nominal weights only.

### Sample Estimates

Let $\varvec{X}_{1},\ldots ,\varvec{X}_{n}\sim F$ be *n* iid vectors of ratings. Then there is a single natural sample estimator of $D_{d}$, namely

2.10 $$\begin{aligned} \hat{D}_{d}=n^{-1}\sum _{i=1}^nD_{d}(\varvec{x}_{i}). \end{aligned}$$

There are, however, two natural estimators of the Cohen-type chance disagreement: one them a *V*-statistic (Lee, 2019, Chapter 4.2) and the other a *U*-statistic (Lee, 2019, Chapter 1),

2.11 $$\begin{aligned} \hat{C}_{d} = n^{-g}\sum _{i_{1},\ldots ,i_{g}}C_{d} (\varvec{x}_{i_{1}},\ldots ,\varvec{x}_{i_g})\quad \text {and}\quad \hat{C}_{d}^{u}=\left( {\begin{array}{c}n\\ g\end{array}}\right) ^{-1}\sum _{i_{1}, \ldots ,i_{g}}C_{d}(\varvec{x}_{i_{1}},\ldots , \varvec{x}_{i_{g}}), \end{aligned}$$

where the first estimator runs over all combinations with repetitions of $i_{1},i_{2},\ldots ,i_{g}$ and the second estimator runs over the unordered combinations $i_{1}<i_{2}<\ldots <i_{g}$. Using the basic results of *U*-statistics (Lee, 2019, Chapter 1), we see that $C_{d}^{u}$ is the unique minimum-variance unbiased estimator of $C_{d}$, which makes it attractive from a theoretical point of view. However, from a well-known correspondence between *U*-statistics and *V*-statistics, the asymptotic distributions of $\hat{C}_{d}$ coincide with the asymptotic distribution of $\hat{C}_{d}^{u}$ (Lee, 2019, Chapter 4, Theorem 1), so the choice between $\hat{C}_{d}$ and $\hat{C}_{d}^{u}$ barely matters when *n* is sufficiently large.

Likewise, there are two natural estimators of the Fleiss-type weighted chance agreement,

2.12 $$\begin{aligned} \hat{F}_{d} = n^{-g}\sum _{i_{1},\ldots ,i_{g}}F_{d}(\varvec{x}_{i_{1}},\ldots , \varvec{x}_{i_{g}})\quad \text {and}\quad \hat{F}_{d}^{u} =\left( {\begin{array}{c}n\\ g\end{array}}\right) ^{-1}\sum _{i_{1},\ldots ,i_{g}}F_{d}(\varvec{x}_{i_{1}},\ldots , \varvec{x}_{i_{g}}), \end{aligned}$$

where the index sets are described above.

Now, we can define two sample variants of Cohen’s kappa (Fleiss’s kappa), depending on which one of $\hat{C}_{d}$ ($\hat{F}_{d}$) and $\hat{C}_{d}^{u}$ ($\hat{F}_{d}^{u}$) we choose to use. These are $\hat{\kappa }_{d}=1-\hat{D}_{d}/\hat{C}_{d}$ and $\hat{\kappa }_{d}^{u}=1-\hat{D}_{d}/\hat{C}_{d}^{u}$ for Cohen’s kappa and $\hat{\pi }_{d}=1-\hat{D}_{d}/\hat{F}_{d}$ and $\hat{\pi }_{d}^{u}=1-\hat{D}_{d}/\hat{F}_{d}^{u}$ for Fleiss’s kappa. The definition of the sample Cohen’s kappa (Cohen, 1968) agrees with $\hat{\kappa }_{d}$, not with $\hat{\kappa }_{d}^{u}$. Likewise, the sample Fleiss’s kappa has a definition agreeing with $\hat{\pi }_{d}$ (Fleiss, 1971). Moreover, due to the possibility of binning data, $\hat{\pi }_{d}$ and $\hat{\kappa }_{d}$ are faster to compute when the data is not continuous. Since the estimators are asymptotically equivalent in any case, we will stick to the *V*-statistics $\hat{\kappa }_{d}$ and $\hat{\pi }_{d}$for estimation, but use the *U*-statistic form when deriving limit distributions. We note that, since we need to compute strictly fewer combinations, $\hat{\kappa }_{d}^{u}$ and $\hat{\pi }_{d}^{u}$ are faster to compute when the data is continuous, which may be useful in some settings.

## Fréchet Variances for *g*-Wise Agreement Coefficients

The most popular measures of agreement are defined only for $g=2$. It is easy to find reasonable disagreement measures in this case, as one can draw on the extensive literature on norms and distances. The $l_{p}$ distances are the obvious choices, but there are many unexplored options, such as the Huber loss (Huber, 1964) and the LINEX loss (Varian, 1975).

In the setting of Hubert’s kappa and the Schuster–Smith coefficient, we have $g=R$, and it is not that easy to find reasonable disagreement functions anymore. The disagreement function used in Hubert’s kappa, $d(x_{1},\ldots ,x_{R})=1-1[x_{1}=\cdots =x_{R}]$, will penalize any number of discordant ratings equally, yielding the often undesirable outcome that most sets of ratings will be in complete disagreement. But there are less sensitive ways to count nominal disagreements. Consider the case of 10 raters with three ratings on an ordinal scale from 1–3, with 7 raters giving rating 1, 2 giving rating 2, and 1 giving rating 3. Then Hubert’s disagreement rating is 1, as the rating vector is not constant, and the pairwise disagreement is 46/100. But it sounds reasonable to pick the modal rating (in this case 1) and then report the number of raters that disagree with it, divided by the number of raters. In this case, the number of raters disagreeing with the modal rating is 3, and the “modal” disagreement equals 3/10.

Sometimes we wish to aggregate numerical ratings instead of categorical ratings. Consider the above case again but with the median (which is 1) instead of the mode. It is well known that the median of a vector *x* is equal to ${{\,\textrm{argmin}\,}}_{\mu }\frac{1}{R}\sum _{r=1}^R|x_{r}-\mu |$, so $\min _{\mu }\frac{1}{R}\sum _{r=1}^R|x_{r}-\mu |$ (mean absolute deviation from the median) appears to be a reasonable measure of the mean disagreement when we use the median as the aggregation method. The resulting mean disagreement of the previous example is $\min _{\mu }\frac{1}{R}\sum _{r=1}^R|x_{r}-\mu |=\frac{1}{10} \sum _{r=1}^{10}|x_{r}-1|=4/10$.

The “modal” and “median” disagreement measures are instances of an intuitive generalization of the variance called the *Fréchet variance* (Dubey and Müller, 2019). Let *l* be a distance function satisfying $l(x,y)\ge 0$ and $l(x,x)=0$, and let $A=\{x_{1},x_{2},\ldots ,x_{R}\}$ be a set of points. The sample *Fréchet mean* of *A* is defined as the (not necessarily unique) point $\mu _{l}$ that minimizes the sum of distances to all points in *A*, that is,^4^

3.1 $$\begin{aligned} \mu _{l}[A]={{\,\textrm{argmin}\,}}_{\mu }\sum _{r=1}^{R}l(\mu ,x_{r}). \end{aligned}$$

Similarly, the sample *Fréchet variance* on *A* with distance function *l* is

3.2 $$\begin{aligned} V(l)[A]=\min _{\mu }\sum _{r=1}^{R}\frac{1}{R}l(\mu ,x_{r}) =\sum _{r=1}^{R}\frac{1}{R}l(\mu _{l}[A],x_{r}). \end{aligned}$$

The Fréchet mean (Fréchet, 1948) is a generalization of centroids to arbitrary distance functions *l*; likewise, the Fréchet variance is a generalization of dispersion to any such distance function. They are best understood through a decision-theoretic lens: The Fréchet mean of *A* represents your best guess of the true classification or value of an item according to the distance *l*; the Fréchet variance *V*(*l*) is the decision-theoretic risk associated with the choice. See Cooil and Rust (1994) for an investigation of a closely related idea in the context of agreement measures.

Define the *g*-dimensional disagreement based on *l* as

3.3 $$\begin{aligned} d(\varvec{x}_{1},\ldots ,\varvec{x}_{g})=V(l) [\{\varvec{x}_{1},\ldots ,\varvec{x}_{g}\}]. \end{aligned}$$

The most important distance functions are:

(i) $d_{0}(x,y)=1[x\ne y]$. Generalizes the nominal distance. If the data are categorical, the Fréchet mean $\mu _{d}$ equals the mode, and the Fréchet variance equals the percentage of observations different from the mode. If we are dealing with vector-valued data with *I* elements each, it might be preferable to use $I^{-1}\sum _{i=1}^{I}1[x_{i}\ne y_{i}]$ instead, as it counts each dimension of the nominal data separately.
(ii) $d_{1}(x,y)=||x-y||_{1}$. For scalar ratings, the Fréchet mean is equal to the sample median. The Fréchet variance equals the sample mean absolute deviation from the median, i.e., $\frac{1}{R}\sum _{r=1}^{R}|x_{r}-\mu _d|$, where $\mu _d$ is the sample median.
(iii) $d_{2}^{2}(x,y)=||x-y||_{2}^{2}$. For scalar ratings, the Fréchet mean is equal to the sample mean $\mu _d=\frac{1}{R}\sum _{r=1}^{R}x_{r}$, and the Fréchet variance is equal to the biased sample variance of $\{x_{1},x_{2},\ldots ,x_{R}\}$, that is, $\frac{1}{R}\sum _{r=1}^{R}(x_{r}-\mu _d)^{2}$.
(iv) $d_{2}(x,y)=||x-y||_{2}$. For vector-valued data, the Fréchet mean has no simple formula, but is known as the *geometric median*. If the data is scalar, $d_{2}=d_{1}$, which implies that the Fréchet mean equals the median, hence the name. There is an extensive literature on the geometric median; see, e.g., Drezner et al. (2002) for an overview and Cohen et al. (2016) for how to compute it. When the ratings are vector-valued, the geometric median is far more computationally expensive than the Fréchet mean based on $||x-y||_{2}^{2}$.

For any $p\in [0,\infty ]$ and pair of vectors $x_{1},x_{2}$, we have the following (proved in Appendix, Sect. 6):

3.4 $$\begin{aligned} V(d_{p})[x_{1},x_{2}]=\frac{1}{2}d_{p}(x_{1},x_{2}),\quad V(d_{p}^{p})[x_{1},x_{2}]=\frac{1}{2^{p}}d_{p}^{p}(x_{1},x_{2}). \end{aligned}$$

It follows that $\kappa _{d_{p}}=\kappa _{V(d_{p})}$ and $\kappa _{d_{p}^{p}}=\kappa _{V(d_{p}^{p})}$ when we are dealing with pairwise agreement. Thus, the Fréchet variances generalize the pairwise agreement for these distances to *g*-wise coefficients. But be aware that the particular case of $V(d_{2}^{2})$ constitutes a trivial generalization, as it can be shown that the kappas do not vary with *g* when using the quadratic Fréchet variance $V(d_{2}^{2})$. It follows that $\kappa _V(d_2^2)$ equals the concordance coefficient for every *g*.

### Example 1

Suppose you have $R=5$ raters and 4 items, with ratings (1, 1, 2, 1, 1), (1, 2, 3, 2, 2), (2, 1, 1, 1, 1), (2, 3, 4, 4, 5). The Fréchet means using the distance $|x-y|$ equals the sample medians 1, 2, 1, 4. The Fréchet variances are $V(d_{1})=(0.2,0.4,0.2,0.8)$. To calculate the sample Cohen’s kappa with $d=V(d_{1})$, we first find the mean disagreement $\overline{V(d_{1})}=0.4$ (2.10), then the mean Cohen disagreement, which is $\approx 0.73$ (2.11). Thus, Cohen’s kappa is $1-0.4/0.73=0.45$.

We believe the most useful distance measures will typically be $$d_{0}$$ for categorical data and $$d_{1}$$ for ordinal data, both using $$g=R$$. The quadratic distance $$d_{2}^{2}$$ could be used for ordinal data as well, but is harder to justify, as it is usually not obvious why we would be interested in the squared distance between two observations rather than just the distance itself. The distances $$d_{p},p\in (1,\infty ]$$, with $$d_{2}$$ included, are even harder to recommend, as they do not work in a coordinatewise manner for vector data. In any case, it seems most reasonable to go with the *R*-wise variants of these distance measures, as they make use of all the available information, but the *g*-wise agreement coefficients ($$g<R)$$ do not.

### Example 2

In the paper introducing what is now called Fleiss’s kappa, Fleiss (1971) discussed an example involving 5 different types of diagnoses, $n=30$ patients, and $R=6$ psychiatrists. The data were originally from Sandifer et al. (1968), but Fleiss removed some ratings to make the design rectangular. We use this data to illustrate the difference between Hubert’s kappa and the Fréchet variances when applied to nominal data with $g=R$.

Hubert’s kappa is $\pi =0.166$ while Fleiss’ kappa using $V(d_0)$ is $\pi =0.486$. The substantial difference suggests that a sizeable number of rating vectors contain at least one rating that disagrees with the others. Table 2 summarizes the relevant aspects of the data. The maximal agreement row could potentially go from 1 to 6, but the smallest number of raters agreeing on the classification of an item in this data set is 3. The count row counts the number of rows with the corresponding maximal agreements and distances. According to the Hubert distance, the raters disagree a lot, since only 5 items have a disagreement of 0 and the rest a disagreement of 1. On the other hand, $V(d_{0})$ results in a much smaller overall disagreement, with all disagreements smaller than the maximum of 1.

**Table 2:** Maximal agreement for the data of Fleiss (1971).

Maximal agreement*	3	4	5	6
Count	8	10	7	5
Distance ($V(d_{0})$)	1/2	1/3	1/6	0
Distance (Hubert)	1	1	1	0

*The largest number of raters that agree on the classification of an item. Both $V(d_{0})$ and Hubert’s distance depend only on this when $g=R$.

## Inference

### Limit Theory Using *U*-Statistics

Let $\varvec{X}_{1},\ldots ,\varvec{X}_{n}$ be independently and identically distributed and $\psi (x_{1},\ldots ,x_{k})$ be a symmetric function. A *U*-statistic of order *k* with kernel $\psi$ is

4.1 $$\begin{aligned} U_{n}=\left( {\begin{array}{c}n\\ k\end{array}}\right) ^{-1}\sum _{i_{1},\ldots ,i_{k}} \psi (\varvec{X}_{i_{1}},\ldots ,\varvec{X}_{i_{k}}), \end{aligned}$$

where the sum extends over all *k*-dimensional tuples satisfying $1\le i_{1}<i_{2}<\cdots \le n$.

The theory of *U*-statistics was established by Hoeffding (1992); for an introduction, see, e.g., Chapter 6.1 of Lehmann (2004), Chapter 5 of Serfling (1980), or the textbook of Lee (2019). These references handle *U*-statistics where the $X_{i}$s are real-valued, but their results, including the simple results below, hold for vector-valued $X_{i}$s as well (Korolyuk and Borovskich, 2013).

The weighted chance agreement of Fleiss-type (Cohen-type) is a *U*-statistic with kernel $$F_{d}$$ ($$C_{d})$$, of order *g*. The disagreement is a *U*-statistic with kernel $$D_{d}$$, which has order 1. To find the asymptotic variance of the kappas, we will use formulas for the asymptotic covariance of *U*-statistics. Let $$U_{1n}$$ and $$U_{2n}$$ be two *U*-statistics of *n* observations with symmetric kernel functions $$\psi _{1}$$, $$\psi _{2}$$ of dimensions $$k_{1}$$ and $$k_{2}$$. Define

$$\begin{aligned} \sigma _{1}^{2}= & {} {{\,\textrm{Var}\,}}(E[\psi _{1}(\varvec{X}_{1},\ldots , \varvec{X}_{k_{1}})\mid \varvec{X}_{1})]),\\ \sigma _{12}= & {} {{\,\textrm{Cov}\,}}(E[\psi _{1}(\varvec{X}_{1},\ldots ,\varvec{X}_{k_{1}}) \mid \varvec{X}_{1})],E[\psi _{2}(\varvec{X}_{1}, \ldots ,\varvec{X}_{k_{2}})\mid \varvec{X}_{1})]). \end{aligned}$$

Then we have $n{{\,\textrm{Cov}\,}}(U_{1n},U_{2n})\rightarrow k_{1}k_{2}\sigma _{12}$ and $n{{\,\textrm{Var}\,}}(U_{1n})\rightarrow k_{1}^{2}\sigma _{1}^{2}$ (Lee, 2019, Theorem 2, p. 76)). It is also possible to calculate the exact covariances, which could potentially make the asymptotic variances for the kappas perform better. See Appendix, Sect. 6 for the formula for the exact covariance (Lee, 2019, Theorem 2, p. 17)).

#### Lemma 1

Define the parameter vectors $\varvec{p}=(D_{d},C_{d},F_{d})$ and $\hat{\varvec{p}}=(\hat{D}_{d},\hat{C}_{d},\hat{F}_{d})$. Then $\sqrt{n}(\hat{\varvec{p}}-\varvec{p}){\mathop {\rightarrow }\limits ^{d}}N(0,\Sigma )$, where $\Sigma$ is the covariance matrix with elements

$$\begin{aligned} \sigma _{11}= & {} \sigma _{D}^{2}= & {} {{\,\textrm{Var}\,}}D_{d}(\varvec{X}_{1})&,\quad&\sigma _{12}= & {} \sigma _{CD}= & {} g{{\,\textrm{Cov}\,}}(\mu _{dC}(\varvec{X}_{1}),D_{d}(\varvec{X}_{1})),\\ \sigma _{22}= & {} \sigma _{C}^{2}= & {} g^{2}{{\,\textrm{Var}\,}}\mu _{dC}(\varvec{X}_{1})&,\quad&\sigma _{13}= & {} \sigma _{FD}= & {} g{{\,\textrm{Cov}\,}}(\mu _{dF}(\varvec{X}_{1}),D_{d}(\varvec{X}_{1})),\\ \sigma _{33}= & {} \sigma _{F}^{2}= & {} g^{2}{{\,\textrm{Var}\,}}\mu _{dF}(\varvec{X}_{1})&,\quad&\sigma _{23}= & {} \sigma _{CF}= & {} g{{\,\textrm{Cov}\,}}(\mu _{dC}(\varvec{X}_{1}),\mu _{dF}(\varvec{X}_{1})). \end{aligned}$$

Here the variable $\mu _{dC}(\varvec{X}_{1})$, and $\mu _{dF}(\varvec{X}_{1})$ are defined as

$$\begin{aligned} \mu _{dC}(\varvec{X}_{1})=E[C_{d}(\varvec{X}_{1}, \ldots ,\varvec{X}_{g})\mid \varvec{X}_{1}] \quad \mu _{dF}(\varvec{X}_{1}) =E[F_{d}(\varvec{X}_{1},\ldots ,\varvec{X}_{g}) \mid \varvec{X}_{1}]. \end{aligned}$$

The form of the covariance matrix follows from the remarks preceding the lemma. Asymptotic normality follows from a general theorem about asymptotic normality of *U*-statistics, see, e.g., Theorem 2 of Lee (2019, p. 76).

We want to use Lemma 1 to find the limit distribution of the generalized Cohen’s kappa and Fleiss’s kappa. To this end, recall the multivariate delta method (see, e.g., Lehmann, 2004, Theorem 5.2.3). Let $f:\mathbb {R}^{k}\rightarrow \mathbb {R}$ be continuously differentiable at $\theta$ and suppose that $\sqrt{n}(\hat{\theta }-\theta ){\mathop {\rightarrow }\limits ^{d}}N(0,\Sigma )$. Then

4.2 $$\begin{aligned} \sqrt{n}[f(\hat{\theta })-f(\theta )]{\mathop {\rightarrow }\limits ^{d}}N(0,\nabla f(\theta )^{T}\Sigma \nabla f(\theta )), \end{aligned}$$

where $\nabla f(\theta )$ denotes the gradient of *f* at $\theta$.

In the case of Cohen’s kappa and Fleiss’s kappa, we find that

4.3 $$\begin{aligned} \nabla \kappa _{d}= & {} \frac{1}{C_{d}}\left( -1,\frac{D_{d}}{C_{d}}\right) , \quad \nabla \pi _{d}=\frac{1}{F_{d}}\left( -1,\frac{D_{d}}{F_{d}}\right) . \end{aligned}$$

Using some algebra, the expressions for the asymptotic variances follow from Lemma 1 and the above gradients.

#### Proposition 1

Then Cohen’s kappa $\hat{\kappa }_{d}$ and Fleiss’s kappa $\hat{\pi }_{d}$ are asymptotically normal, and their asymptotic variances are

4.4 $$\begin{aligned} \sigma _{\kappa }^{2}= & {} \sigma _{D}^{2}\frac{1}{C_{d}^{2}}-2\sigma _{CD} \frac{D_{d}}{C_{d}^{3}}+\sigma _{C}^{2}\frac{D_{d}^{2}}{C_{d}^{4}}, \nonumber \\ \sigma _{\pi }^{2}= & {} \sigma _{D}^{2}\frac{1}{F_{d}^{2}}-2\sigma _{FD}\frac{D_{d}}{F_{d}^{3}} +\sigma _{F}^{2}\frac{D_{d}^{2}}{F_{d}^{4}}. \end{aligned}$$

These results are also valid for $\hat{\kappa }_{d}^{u}$ and $\hat{\pi }_{d}^{u}$. Since the sample Krippendorff’s alpha (see note below) is equal to $\hat{\alpha }_{d}=\hat{\pi }_{d}+\frac{1}{2Rn}(1-\hat{\pi }_{d})$, it is also asymptotically normal with asymptotic variance $\sigma _{\pi }^{2}$.

With $g=2$ and a finite number of categories, Schouten (1980) derived $\sigma _{\pi }^{2}$, while Schouten (1982) and O’Connell and Dobson (1984) derived $\sigma _{\kappa }^{2}$. The result for Krippendorff’s alpha is, to our knowledge, new.

**A brief aside on Krippendorff’s alpha** Krippendorff’s alpha (Krippendorff, 1970) is an agreement coefficient especially popular in content analysis (Krippendorff, 2018). It has no population definition, but its sample definition equals $\hat{\alpha }_{d}=\hat{\pi }_{d}+\frac{1}{N}(1-\hat{\pi }_{d})$ (the total sample size *N* equals 2*Rn* in the case of a rectangular design); see Proposition 3 in Appendix for a justification. For this reason, all of the results about the limit of $\hat{\pi }_{d}^{u}$ apply to Krippendorff’s alpha as well, as it is an asymptotically equivalent estimator of $\pi _{d}$. Note, however, that Krippendorff (2018) emphasizes the use of non-rectangular designs, and the limit results in the preceding section do not hold for such study designs.

### Estimating the Variances

The unknown quantities $\hat{D}_{d}$, $\hat{C}_{d}$, and $\hat{F}_{d}$ can be estimated using their sample counterparts. The variances and covariances can be estimated using the empirical (co)variances of the estimated $\hat{\mu }$s. These have formulas

4.5 $$\begin{aligned} \hat{\mu }_{d}(\varvec{x}_{i})= & {} D_{d}(\varvec{x}_{i}),\nonumber \\ \hat{\mu }_{dC}(\varvec{x}_{i})= & {} n^{-(g-1)}\sum _{i_{1},\ldots ,i_{g-1}}C_{d}(\varvec{x}_{i}, \varvec{x}_{i_{1}},\ldots ,\varvec{x}_{i_{g-1}}),\nonumber \\ \hat{\mu }_{dF}(\varvec{x}_{i})= & {} n^{-(g-1)}\sum _{i_{1},\ldots ,i_{g-1}}F_{d}(\varvec{x}_{i}, \varvec{x}_{i_{1}},\ldots ,\varvec{x}_{i_{g-1}}), \end{aligned}$$

where the index sets run over all combinations with repetitions of $(i_{1},i_{2},\ldots ,i_{g-1})$.

Observe that estimating $\hat{\mu }_{dC}$ and $\hat{\mu }_{dF}$ directly is computationally very expensive, especially when done without binning, which cannot be done with continuous data. The obvious computation of all $\hat{\mu }_\text {dC}$ requires a number of operations on the order of $n^{g-1}$, which is prohibitively expensive for large *n* and *g*. However, there are few applications of agreement measures with very large *n* and *g*, so this should not be a serious problem in practice. We note that less computationally demanding procedures are possible for the quadratic Fréchet variance $V(d_2^2)$, as it can be shown that its associated kappas are invariant under *g*. Thus, we may use the computationally very effective methods for the concordance coefficient outlined by, e.g., Carrasco and Jover (2003).

From the definitions of $\hat{D}_{d},\hat{C}_{d}$, and $\hat{F}_{d},$ (4), we quickly deduce that $\overline{\hat{\mu }_{d}}=\hat{D}_{d}$, $\overline{\hat{\mu }_{dC}}=\hat{C}_{d}$ and $\overline{\hat{\mu }_{dF}}=\hat{F}_{d}$. Using this fact, we can define the estimators

$$\begin{aligned} \hat{\sigma }_{C}^{2}=\frac{g^2}{n-1}\sum _{i=1}^{n}(\hat{\mu }_{dC} (\varvec{x}_{i})-\hat{C}_{d})^{2},\quad \hat{\sigma }_{CD}^{2} =\frac{g}{n-1}\sum _{i=1}^{n}(\hat{\mu }_{dC}(\varvec{x}_{i}) -\hat{C}_{d})(\hat{\mu }_{d}(\varvec{x}_{i})-\hat{D}_{d}), \end{aligned}$$

and $\hat{\sigma }_{D}^{2}=\frac{1}{n-1}\sum _{i=1}^{n}(\hat{\mu }_{d} (\varvec{x}_{i})-\hat{D}_{d})^{2}$. Moreover, we can estimate $\hat{\sigma }_{F}^{2}$ and $\hat{\sigma }_{FD}^{2}$ in the same way, substituting $\hat{\mu }_{dF}$ for $\hat{\mu }_{dC}$. Using the formulas for the theoretical variances (4.4), we find the estimators

4.6 $$\begin{aligned}{} & {} \hat{\sigma }_{\kappa }^{2}=\hat{\sigma }_{D}^{2}\frac{1}{\hat{C}_{d}^{2}} -2\hat{\sigma }_{CD}\frac{\hat{D}_{d}}{\hat{C}_{d}^{3}} +\hat{\sigma }_{C}^{2}\frac{\hat{D}_{d}^{2}}{\hat{C}_{d}^{4}}, \end{aligned}$$

4.7 $$\begin{aligned}{} & {} \hat{\sigma }_{\pi }^{2}=\hat{\sigma }_{D}^{2}\frac{1}{\hat{F}_{d}^{2}} -2\hat{\sigma }_{FD}\frac{\hat{D}_{d}}{\hat{F}_{d}^{3}}+\hat{\sigma }_{F}^{2} \frac{\hat{D}_{d}^{2}}{\hat{F}_{d}^{4}}. \end{aligned}$$

The variance estimator $\hat{\sigma }_{\pi }^{2}$ coincides with that of Gwet (2021, equation 4) in the case of nominal weights; see Appendix (Sect. 6) for a proof sketch.

### Improving Approximate Normality with the Arcsine and Fisher Transforms

It is well known that the *Fisher transform* (Fisher, 1915) improves the inference for the correlation coefficient. If *r* is the sample correlation, ${{\,\textrm{artanh}\,}}(r)=\frac{1}{2}\log [(1+r)/(1-r)]$ has approximately the same variance for most *r*, and its distribution is closer to normal than that of the untransformed *r*, especially when the population correlation is close to $\pm 1$. This transform makes sense outside the world of correlations; for instance, Lin (1989) used the Fisher transform to improve the normality of the quadratically weighted Cohen’s kappa.

The arcsine is another reasonable transformation of $\hat{\kappa }_{d}$ and $\hat{\pi }_{d}$. The arcsine is the inverse of the sine function and is defined as $\arcsin x=\int 1/\sqrt{1-x^{2}}\textrm{d}x$. In ecology (Warton and Hui, 2011), the arcsine transformation denotes $\arcsin \sqrt{p}$, where *p* is a probability. We do not take square root, however, as $\hat{\kappa }_{d}$ and $\hat{\pi }_{d}$ can be negative.

Calculating the limiting variance of $\arcsin \hat{\kappa }_{d}$ and $\arcsin \hat{\pi }_{d}$ requires an additional application of the delta method (4.2). Using that $\frac{\textrm{d}}{\textrm{d}x}\arcsin (x)=1/\sqrt{1-x^{2}}$ and $\frac{\textrm{d}}{\textrm{d}x}{{\,\textrm{artanh}\,}}(x)=1/(1-x^{2})$, we find

4.8 $$\begin{aligned} \sqrt{n}(\arcsin \hat{\kappa }_{d}-\arcsin \kappa _{d})\rightarrow & {} N(0,(1-\kappa _{d}^{2})^{-1}\sigma _{\kappa }^{2}), \end{aligned}$$

4.9 $$\begin{aligned} \sqrt{n}({{\,\textrm{artanh}\,}}\hat{\kappa }_{d}-{{\,\textrm{artanh}\,}}\kappa _{d})\rightarrow & {} N(0,(1-\kappa _{d}^{2})^{-2}\sigma _{\kappa }^{2}). \end{aligned}$$

Expressions for $\hat{\pi }_{d}$ can be found by swapping $\kappa _{d}$ for $\pi _{d}$ and $\sigma _{\kappa }^{2}$ for $\sigma _{\pi }^{2}$.

#### Example 3

**Fig. 1:**
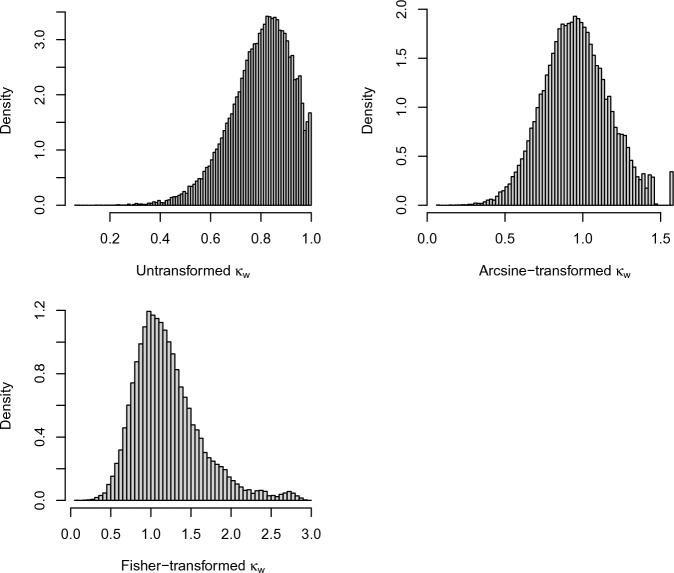
Simulated sampling distribution of $\hat{\kappa }_{d}$ for quadratic weights using three transformations, $n=20, R=3$. The simulation setup is described in Example 3. The arcsine transform makes the sampling distribution closest to the normal distribution.

This example illustrates that the arcsine and Fisher transforms may make the sampling distribution closer to the normal distribution. Let the number of raters be $R=3$, the disagreement function be quadratic (with $g=2$), and the number of items be $n=20$. There are five categories and the true classification of an item is one of $\{1,2,3,4,5\}$ with probability 1/5 each. Every rater knows the true classification of an item with probability 0.9. If they do not know the correct classification, they will guess a classification from $\{1,2,3,4,5\}$ uniformly at random. One can show that the population value of the quadratically weighted Cohen’s kappa is 0.816 under these circumstances, following the arguments of Perreault and Leigh (1989). We simulate the value of $\hat{\kappa }_{d}$ a total of $N=50,000$ times and transform them using the identity transform, the arcsine transform, and the Fisher transform. The results are shown in Fig. 1. The arcsine transform appears to bring the sampling distribution of $\hat{\kappa }_{d}$ closer to the normal distribution, with the Fisher transform also improving normality quite a bit.

## Confidence Intervals

Using the methodology we have developed, we can easily construct confidence intervals for the agreement coefficients.

We describe our three confidence interval constructions only for Cohen’s kappa, as the intervals using Fleiss’ kappa can be found by replacing every instance $\hat{\kappa }_{d}$ with $\hat{\pi }_{d}$ and $\hat{\sigma }_{\kappa }^{2}$ with $\hat{\sigma }_{\pi }^{2}$. We use the two-sided *t*-distribution-based confidence intervals with nominal level $1-\alpha =0.95$. Let *c* be the $(1-\alpha /2)$-quantile of the *t* distribution with $n-1$ degrees of freedom. The basic interval is

5.1 $$\begin{aligned} {[}\hat{\kappa }_{d}-c\hat{\sigma }_{\kappa }/\sqrt{n-1}, \hat{\kappa }_{d}+c\hat{\sigma }_{\kappa }/\sqrt{n-1}], \end{aligned}$$

where $\hat{\sigma }_\kappa$ is the estimated variance described in equation (4.6).

The arcsine interval replaces the basic limits with

5.2 $$\begin{aligned} \sin \left( \arcsin \hat{\kappa }_{d}\pm c(1-\hat{\kappa }_{d}^{2})^{-1/2}\hat{\sigma }_{\kappa }/ \sqrt{n-1}\right) , \end{aligned}$$

where $(1-\hat{\kappa }_{d}^{2})^{-1}\hat{\sigma }_{\kappa }^{2}$ is the asymptotic variance of $\arcsin \hat{\kappa }_{d}$ (4.8). The Fisher interval uses the area hyperbolic tangent,

5.3 $$\begin{aligned} \tanh \left( {{\,\textrm{artanh}\,}}\hat{\kappa }_{d}\pm c(1-\hat{\kappa }_{d}^{2})^{-1}\hat{\sigma }_{\kappa }/ \sqrt{n-1}\right) , \end{aligned}$$

where $(1-\hat{\kappa }_{d}^{2})^{-2}\hat{\sigma }_{\kappa }^{2}$ is the asymptotic variance of ${{\,\textrm{artanh}\,}}\hat{\kappa }_{d}$ (4.9).

Using the methodology just described, we can calculate confidence intervals for the Fleiss (1971) data of Example 2.

### Example 4

(Ex. 2 cont.) Using the data of Fleiss (1971), we calculate arcsine confidence intervals for the *g*-wise Fleiss’s kappa. The raters are not the same for all items, but it seems plausible to assume that the ratings are exchangeable given the item. The diagnoses are essentially categorical in nature; hence, we will only consider $V(d_{0})$ and Hubert’s disagreement function. The results are shown in Table 3. We see that the agreement coefficients agree when $g=2$, as both $V(d_{0})$ and Hubert’s disagreement function equals the nominal agreement in this case. But the coefficients differ substantially as *g* increases. This is to be expected, as Hubert’s disagreement function measures consensus while $V(d_{0})$ measures the number of observations different from the mode. Observe that $V(d_0)$ is not invariant with respect to *g*, hence it is a proper alternative to the classical Fleiss’s kappa. Moreover, all confidence intervals are of comparable length.

**Table 3:** Confidence intervals for the data of Fleiss (1971) using the arcsine method.

$\varvec{d}$	Fleiss’ kappa								
	$g=2$			$g=3$			$g=6$*		
	$\text {CI}_{l}$	$\text {CI}_{u}$	$\hat{\pi }$	$\text {CI}_{l}$	$\text {CI}_{u}$	$\hat{\pi }$	$\text {CI}_{l}$	$\text {CI}_{u}$	$\hat{\pi }$
$V(d_{0})$	0.314	0.539	0.43	0.388	0.597	0.496	0.366	0.597	0.486
Hubert^†^	0.314	0.539	0.43	0.202	0.458	0.333	0.021	0.308	0.166

*This is Hubert’s kappa when the Hubert disagreement is used.

^†^Hubert disagreement equals the nominal disagreement $V(d_{0})$ when $g=2$.

The preceding example fits best into the context of Fleiss’ kappa, as the identity of the raters are unknown. Moreover, there is no ordinal structure in the data, making the $V(d_1)$ and $V(d_2^2)$ distances unnatural to employ. Our next example concerns the Fréchet variances applied to a case of ordinal data when the identity of the raters are known.

### Example 5

Zapf et al. (2016) studied bootstrap intervals for Fleiss’s kappa and Krippendorff’s alpha using simulations and a case study. Their case study concerned the histopathological assessment of breast cancer and involved ratings performed by $R=4$ senior pathologists and $n=50$ breast cancer biopsies. We apply the arcsine method to calculate confidence intervals and point estimates, displayed in Table 4. We focus on Cohen’s kappa since the same four pathologists rate each cancer biopsy, but we include a column for Fleiss’s kappa when $g=4$ for comparison’s sake. When $g=4$, Cohen’s kappa and Fleiss’s kappa are as good as indistinguishable. As can be verified by using the code in the supplementary material, this happens for the other *g*s as well. It is not generally the case that Fleiss’s kappa and Cohen’s kappa nearly coincide, but it is likely to happen if the marginal ratings are approximately the same for all raters, as is the case in this data set. There is a sizable difference between the disagreement functions, but there is not typically a big difference when changing *g*s, provided we keep the disagreement functions constant. It remains to be seen whether this is common or not. The exception is Hubert’s disagreement function, which decreases quite a bit. (As in the Fleiss (1971) example, this is expected, as the Hubert’s disagreement function is a consensus measure.) Observe that the kappas under the quadratic Fréchet variance $V(d^2_2)$ do not change with *g*, which is always the case.

**Table 4:** Confidence intervals for Zapf et al. (2016) using the arcsine method.

$\varvec{d}$	Cohen’s kappa						Fleiss’ kappa		
	$g=2$			$g=4^{\dagger }$			$g=4$		
	$\text {CI}_{l}$	$\text {CI}_{u}$	$\hat{\kappa }$	$\text {CI}_{l}$	$\text {CI}_{u}$	$\hat{\kappa }$	$\text {CI}_{l}$	$\text {CI}_{u}$	$\hat{\pi }$
$V(d_{0})$	0.453	0.672	0.567	0.475	0.701	0.594	0.466	0.700	0.589
$V(d_{1})$	0.699	0.857	0.784	0.713	0.870	0.798	0.710	0.870	0.797
$V(d_{2}^{2})$	0.834	0.948	0.898	0.834	0.948	0.898	0.834	0.948	0.898
Hubert^†^	0.453	0.672	0.567	0.276	0.565	0.426	0.271	0.564	0.423

^†^Hubert disagreement equals the nominal disagreement $V(d_{0})$ when $g=2$.

### Simulation of Confidence Sets When $g=2$

We include a small simulation study on the performance of confidence sets using two models: A *Perreault–Leigh model* for discrete rating data and a normal model for continuous rating data. For both models, we investigate the following parameters:

(i) **Number of raters** *R*. We use 2, 5, 20, which corresponds to a small, medium, and large selection of raters.
(ii) **Sample sizes** *n*. We use $n=10,40,100$, corresponding to small, medium, and large agreement studies.
(iii) **Disagreement functions**. Nominal disagreement $1[x\ne y]$, quadratic disagreement $(x-y)^{2}$, and absolute value disagreement $|x-y|$.
(iv) **Methods.** A basic interval without transformations, an arcsine-transformed interval, and a Fisher transformed interval.

#### A Perreault–Leigh Model

Perreault and Leigh (1989) discussed a particular model for ratings in which each rated user either knows the correct answer or guesses uniformly at random. Similar models have been used by Gwet (2008); Maxwell (1977), among others; see Moss (2023) for a thorough discussion of such models. We assume there are five categories encoded as $C=\{-2,-1,0,1,2\}$, and the distribution of the true classification distribution is uniform. For each item rated, the *r*th rater knows the correct classification with probability $\sqrt{0.8}$. If not, he guesses, picking a number from *C* uniformly at random. Then $\kappa _{d}=\pi _{d}=0.8$ for all weights and the number of raters, as can be verified by following the arguments of Perreault and Leigh (1989). We run each simulation $N=10,000$ times.

The simulated lengths and coverages for Cohen’s kappa are given in Table 5. Two features stand out in Table 5. First, the confidence intervals have almost indistinguishable lengths and coverages when either *R* or *n* is large. Second, the basic interval has worse coverage than the arcsine and Fisher intervals when *n* is small, with the Fisher interval having coverage slightly closer to nominal than the arcsine interval. However, the better nominal coverage comes at the expense of greater lengths. In particular, for the absolute value weight, the coverage of the arcsine interval is greater than the coverage of the Fisher interval, but its length is shorter! The table for Fleiss’s kappa is similar and can be found in Appendix, Table 8.

**Table 5:** Coverage (first entry) and lengths (second entry) of confidence intervals: Perreault–Leigh model, Cohen’s kappa.

	Method		Perreault–Leigh model, Cohen’s kappa								
			$R=2$			$R=5$			$R=20$		
		*n*	10	40	100	10	40	100	10	40	100
*Weights*											
Nominal	Basic		0.81	**0.96**	**0.96**	0.92	**0.95**	**0.95**	**0.96**	**0.95**	**0.95**
			0.53	0.30	0.18	0.41	0.18	0.11	0.23	0.09	0.06
	Arcsine		**0.98**	**0.95**	**0.95**	**0.97**	**0.95**	**0.95**	**0.95**	**0.95**	**0.95**
			0.73	0.29	0.18	0.43	0.18	0.11	0.23	0.09	0.06
	Fisher		**0.97**	**0.96**	**0.96**	**0.96**	**0.95**	**0.95**	**0.95**	0.94	**0.95**
			0.91	0.32	0.19	0.50	0.19	0.11	0.24	0.09	0.06
Quadratic	Basic		0.65	0.87	0.92	0.84	0.93	**0.95**	0.94	**0.95**	**0.95**
			0.58	0.39	0.26	0.49	0.26	0.16	0.34	0.14	0.08
	Arcsine		0.82	0.89	0.93	0.88	0.94	**0.95**	0.94	**0.95**	**0.95**
			0.78	0.39	0.25	0.55	0.26	0.16	0.34	0.14	0.08
	Fisher		**0.95**	0.91	**0.95**	0.90	0.94	**0.95**	0.93	**0.95**	**0.95**
			0.94	0.44	0.27	0.65	0.27	0.16	0.37	0.14	0.08
Absolute value	Basic		0.80	0.91	0.93	0.90	0.94	**0.95**	**0.95**	**0.95**	**0.95**
			0.55	0.33	0.21	0.44	0.21	0.13	0.27	0.11	0.06
	Arcsine		**0.98**	0.94	**0.95**	0.94	**0.95**	**0.95**	**0.95**	**0.95**	**0.95**
			0.75	0.33	0.21	0.47	0.21	0.13	0.27	0.11	0.06
	Fisher		**0.97**	**0.95**	**0.95**	**0.95**	**0.95**	**0.96**	0.94	**0.95**	**0.95**
			0.93	0.35	0.21	0.55	0.21	0.13	0.28	0.11	0.07

Coverages greater than 0.95 are in bold.

#### Normal Model

In this study, the rating data is distributed according to the multivariate normal $N(0,\Sigma )$, where $\Sigma$ is the $R\times R$ correlation matrix with off-diagonal elements $\Sigma _{r_{i}r_{j}}=\rho$. Since the data is continuous, we study the absolute value disagreement $d_{1}$ and the quadratic disagreement $d_{2}^{2}$ only. The true values are $\kappa _{d_{2}}=\pi _{d_{2}^{2}}=\rho$ and $\kappa _{d_{1}}=\pi _{d_{1}}=1-\sqrt{1-\rho }$. See Appendix (Sect. 6) for details on the computation of these true values. We use $\rho =0.7$, and hence, $\kappa _{d_{2}^{2}}=0.7$ and $\kappa _{d_{1}}=0.45$. We run each simulation $N=1,000$ times.^5^ We note that agreement coefficients are often called concordance coefficients when dealing with continuous data, especially when the quadratic distance is used. Lin’s concordance coefficient (Lin, 1989, 1992) is a prominent example.

The simulated lengths and coverages for Cohen’s kappa are given in Table 6. There is barely any difference between the three confidence interval constructions. Taken together with the results for the Perreault–Leigh model, where the basic interval performs worse than the other two, we would recommend the usage of either the arcsine or Fisher interval. Again, the table for Fleiss’s kappa is very similar and can be found in Appendix (Table 9).

**Table 6:** Coverage (first entry) and lengths (second entry) of confidence intervals: normal model, Cohen’s kappa.

	Cohen’s kappa: normal model										
	Method		$R=2$			$R=5$			$R=20$		
		*n*	10	40	100	10	40	100	10	40	100
*Weights*											
Quadratic	Basic		0.88	0.92	**0.95**	0.91	**0.95**	0.94	0.88	0.93	**0.95**
			0.66	0.32	0.20	0.50	0.23	0.14	0.43	0.20	0.12
	Arcsine		0.88	0.93	**0.95**	0.90	**0.95**	0.94	0.87	0.92	0.94
			0.67	0.32	0.20	0.49	0.23	0.14	0.42	0.20	0.12
	Fisher		0.90	0.94	0.94	0.88	0.94	0.94	0.86	0.92	0.94
			0.70	0.33	0.20	0.51	0.23	0.14	0.43	0.20	0.12
Absolute value	Basic		0.92	0.94	0.94	0.92	0.93	**0.95**	0.87	0.94	0.94
			0.67	0.31	0.19	0.46	0.21	0.13	0.38	0.18	0.11
	Arcsine		0.93	0.94	0.94	0.92	0.93	**0.95**	0.87	0.94	0.94
			0.65	0.31	0.19	0.45	0.21	0.13	0.38	0.18	0.11
	Fisher		0.93	0.94	**0.95**	0.92	0.93	**0.95**	0.86	0.94	0.94
			0.65	0.31	0.19	0.45	0.21	0.13	0.38	0.18	0.11

Coverages greater than 0.95 are in bold.

### Simulation of Confidence Sets when $g\ne 2$

Table 7 contains simulations from the Perreault–Leigh model (Sect. 5.1.1) with $N=1000$ repetitions and $R=5$ raters using the Fréchet variances $V(d_{0})$, $V(d_{1})$, and Hubert’s disagreement function. We drop $V(d_{2}^{2})$ since it does not vary with *g*. To save space, we drop the basic confidence interval in the simulation. As before, we show the results only for the Cohen-type disagreement, with the Fleiss-type disagreement relegated to Appendix (Table 10). All coverages are decent, and the coverages and lengths are similar across the board.

**Table 7:** Coverage (first entry) and lengths (second entry) of confidence intervals for *g*-wise coefficients: Perreault–Leigh model, Cohen’s kappa.

	Method		Perreault–Leigh model, Cohen’s kappa								
			$g=3$			$g=4$			$g=5$		
		*n*	10	40	100	10	40	100	10	40	100
*Weights*											
$V(d_{0})$	Arcsine		**0.98**	**0.96**	0.94	**0.97**	**0.95**	0.94	**0.98**	0.93	0.93
			0.41	0.16	0.10	0.39	0.16	0.10	0.38	0.15	0.09
	Fisher		**0.96**	**0.96**	0.94	**0.96**	**0.96**	0.94	**0.96**	0.94	0.94
			0.46	0.17	0.10	0.44	0.16	0.10	0.42	0.15	0.09
$V(d_{1})$	Arcsine		0.94	0.94	**0.95**	0.94	0.94	0.94	0.94	**0.95**	**0.95**
			0.49	0.21	0.13	0.45	0.19	0.11	0.44	0.19	0.11
	Fisher		**0.96**	0.94	**0.95**	**0.95**	**0.95**	**0.95**	**0.96**	**0.95**	**0.95**
			0.55	0.21	0.13	0.51	0.19	0.12	0.51	0.19	0.12
Hubert	Arcsine		**0.98**	**0.95**	**0.96**	**0.98**	**0.95**	**0.96**	**0.98**	**0.95**	**0.95**
			0.52	0.22	0.13	0.62	0.26	0.16	0.71	0.31	0.19
	Fisher		**0.97**	**0.96**	**0.96**	**0.97**	**0.96**	**0.96**	**0.98**	**0.96**	0.94
			0.57	0.22	0.13	0.67	0.27	0.16	0.77	0.31	0.19

Coverages greater than 0.95 are in bold.

## Concluding Remarks

When choosing an agreement coefficient one has to carefully think through exactly what one wishes to measure. The Fréchet variances are attractive because of their interpretation. You measure how much the raters disagree with the generalized mean rater, and then adjust for chance. In the case of nominal data, we measure the disagreement with the modal rater. When dealing with numerical data, we may measure disagreement with the median rater (using the absolute value distance), or the mean rater (using the quadratic distance), or use any other Fréchet variance defined on numeric data.

When dealing with nominal data, we believe that using the Fréchet variance, which measures the distance from the mode, is a reasonable choice. But other options are certainly possible, even when dealing with *g*-wise agreement measures. For example, one could use the entropy instead, with distance measure $d(x_{1},x_{2},\ldots ,x_{g})=-\sum _{i=1}^{g}\frac{\#i}{g}\log \frac{\#i}{g}$, where $\#i$ counts the number of elements in $(x_{1},x_{2},\ldots ,x_{g})$ classified as *i*, which could be useful when the number of raters is finite but large. The topic of how to choose reasonable distance measures for *g*-wise agreement studies has not been thoroughly studied, and there might be options preferable to the Fréchet variances that have not yet been found.

We have only covered rectangular design, where every item is rated by the same number of raters. It is quite easy to generalize the definitions of $\kappa _{d}$ and $\pi _{d}$ to non-rectangular designs, as we have done in Appendix, Sect. 6. But inference appears to be quite difficult, probably requiring additional assumptions for the case of non-exchangeable ratings.

In Sect. 4, we introduced the *U*-statistic-based estimators of $C_d$ and $F_d$, but only used them for theoretical purposes. The *U*-statistic-based estimators may plausibly outperform the classical *V*-statistic-based estimators since they are minimum variance unbiased estimators. It would be interesting to see whether the *U*-statistic-based estimators could outperform the traditional *V*-statistic-based estimators when *n* is small, for example in terms of mean squared error or confidence interval coverage.

The confidence intervals based on the arcsine and Fisher transforms perform better than the basic, untransformed interval. It is unclear which one of these intervals to prefer, but it barely matters when the sample size is sufficiently large. It might be possible to improve all of these intervals. Small-sample corrections to the variance appear feasible, with potential openings in the application of the delta rule and in the calculation of $\Sigma$ of Lemma 1. We have used the arcsine and Fisher transforms to improve approximate normality of $\hat{\kappa }_{d}$ and $\hat{\pi }_{d}$, but this choice is semi-arbitrary. Better variance-stabilizing transformations might be found by inspecting the formula for the variances of $\hat{\kappa }_{d}$ and $\hat{\pi }_{d}$ in Proposition 1. The confidence intervals used in the simulation are only known to be first-order accurate. To make second-order accurate confidence intervals, it would be possible to use the explicit formula for the variances to construct studentized confidence intervals, i.e., bootstrap-*t* intervals (Efron, 1987), which are second-order accurate.

**Fig. 2:**
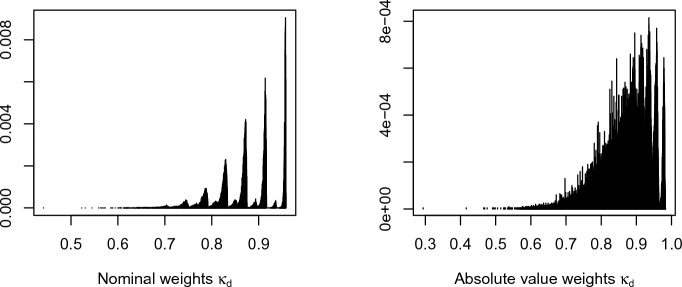
Sample distribution of $\hat{\kappa }_{d}$ for nominal (left) and absolute value (right) weights. Both plots omit a dominating spike at 1. Here $n=20$ and $j=5$, and we use the Perreault–Leigh model (same parameters as in Sect. 5.1) to simulate the data. There were 2573 unique values for the nominal weight and 8790 unique values for the absolute value weight after $N=200{,}000$ simulations.

None of these approaches is guaranteed to help when *n* is small, especially when dealing with categorical data, as the sampling distributions of $\hat{\kappa }_{d}$ and $\hat{\pi }_{d}$ are discrete and highly irregular. For example, consider the sample distribution of the Perreault–Leigh model (Sect. 5.1) when $n=20$ and $R=20$, displayed in Fig. 2. (We omit a dominating spike at 1.) As there are $C=5<\infty$ categories, there is a finite number of possible values for $\hat{\kappa }_{d}$ to take, which is strongly reflected in the plots, especially for the nominal weight.

The superior performance of methods such as the bootstrap-*t* depends on the quantity $\frac{\hat{\theta }-\theta }{{{\,\textrm{se}\,}}}$ being approximately pivotal, that is, approximately the same for all parameters, possibly after applying a transformation. Judging from the plots in Fig. 2, there is no such transformation.

### Supplementary Information

Below is the link to the electronic supplementary material.

Supplementary file 1 (zip 5327 KB)

## Appendix

### Agreement Versus Disagreement

Agreement weighting functions are frequently standardized to guarantee that $w(x_{1},x_{2})\ge 0$, e.g., $w(x_{1},x_{2})=1-|x_{1}-x_{2}|/\max (|x_{1}-x_{2}|)$ for the absolute value weights. Standardization is not necessary, as they do not change the values of $\kappa _{d}$ and $\pi _{d}$ when it is possible (i.e., when $\max (|x_{1}-x_{2}|)<\infty$), and is not defined otherwise. We choose not to use this operation, as it does not change the value of the agreement coefficients in this paper and is impossible to do when the range of $x_{1},x_{2}$ is unbounded.

### Proof of Equivalence Between $V(d_{p})(\varvec{x}_{1},\varvec{x}_{2})$ and $||\varvec{x}_{1}-\varvec{x}_{2}||$

#### Proof

We will show that

$$\begin{aligned} V(d_{p})[\varvec{x}_{1},\varvec{x}_{2}]= \frac{1}{2}||\varvec{x}_{1}-\varvec{x}_{2}||_{p},\quad V(d_{p}^{p})[\varvec{x}_{1},\varvec{x}_{2}]= \frac{1}{2^{p}}||\varvec{x}_{1}-\varvec{x}_{2}||_{p}^{p}. \end{aligned}$$

First, consider the case when $p\ge 1$. Using translation invariance and homogeneity of the norm,

$$\begin{aligned}{} & {} ||x_{1}-\mu ||_{p}+||x_{2}-\mu ||_{p},\\{} & {} \quad = ||x_{1}-\frac{x_{1}+x_{2}}{2}-\mu +\frac{x_{1}+x_{2}}{2}||_{p}+||x_{2}-\frac{x_{1}+x_{2}}{2}-\mu +\frac{x_{1}+x_{2}}{2}||_{p},\\{} & {} \quad = ||\frac{x_{1}-x_{2}}{2}-\nu ||_{p}+||-\frac{x_{1}-x_{2}}{2}-\nu ||_{p},\\{} & {} \quad = ||a-\nu ||_{p}+||a+\nu ||_{p}, \end{aligned}$$

where $$a=\frac{x_{1}-x_{2}}{2}$$ and $$\nu =\mu -\frac{x_{1}+x_{2}}{2}$$.

Observe that

$$\begin{aligned} {{\,\textrm{argmin}\,}}_{\nu }||a+\nu ||_{p}+||a-\nu ||_{p}=0,\quad \text {for all }a \end{aligned}$$

implies $$\mu =\frac{x_{1}+x_{2}}{2}$$.

By the Minkowski inequality,

$$\begin{aligned} 2^{p}||a||^{p}=||a+\nu +a-\nu ||^{p}\le (||a-\nu ||+||a+\nu ||)^{p}. \end{aligned}$$

This is an equality if $||a-\nu ||=||a+\nu ||=||a||$, i.e., when $\nu =0,$ as the left side equals $(||a-\mu ||+||a+\mu ||)^{p}=2^{p}||a||^{p}$. Now it is easy to verify that $V(d_{p})$ and $V(d_{p}^{p})$ have the claimed form; just substitute the value $\mu =\frac{x_{1}+x_{2}}{2}$ into the formula for the Fréchet variance, $\frac{1}{2}(||x_{1}-\mu ||_{p}+||x_{2}-\mu ||_{p})$.

When $0<p<1$, the function $\mu \mapsto ||x_{1}-\mu ||_{p}+||x_{2}-\mu ||_{p}$ is stepwise concave on $[-\infty ,x_{1}]$,$[x_{1},x_{2}]$, and $[x_{2},\infty )$; hence, its minimum is either $x_{1},x_{2}$, or both. It is clear that both $x_{1}$ and $x_{2}$ maps to $||x_{1}-x_{2}||_{p}$; hence, both are Fréchet means. The case $p=0$ is obvious and omitted. $\square$

### True Values in the Normal Simulation

We give a brief explanation why the true values of $\kappa _{d}$ and $\pi _{d}$ are 0.8 for the quadratic weights and $1-\sqrt{0.2}$ for the absolute value weights.

First notice that, since the marginals of $X_{r_{1}}$ and $X_{r_{2}}$ are equal for all $r_{1},r_{2}$, we have that $\kappa _{d}=\pi _{d}$. Moreover, we can ignore the number of raters, since the pairwise distribution do not depend on them. Then, from standard theory about the multivariate and folded normal, we find that

$$\begin{aligned} E(|X_{r_{1}}-X_{r_{2}}|)=2\sqrt{\frac{1-\rho }{\pi }},\quad E(|X_{r_{1}}-X_{r_{2}}|^{2})=2(1-\rho ). \end{aligned}$$

Let $X'_{r_{1}}$be a copy of $X_{r_{1}}$ that is independent of $X_{r_{2}}$. Then $E(|X'_{r_{1}}-X_{r_{2}}|)=2/\sqrt{\pi }$ and $E(|X'_{r_{1}}-X_{r_{2}}|^{2})=2$. Now rewrite the kappas using disagreement instead of agreement. Use the fact that $(p_{wa}-p_{fa})/(1-p_{fa})=1-d_{wa}/d_{fa}$, where $d_{wa}=1-E(w(X_{r_{1}},X_{r_{2}}))$ and $d_{fa}=1-E(w(X'_{r_{1}},X_{r_{2}}))$, where $X'_{r_{1}}$is a copy of $X_{r_{1}}$ that is independent of $X_{r_{2}}$.

Thus, $\kappa _{d}=\pi _{d}=1-E(|X_{r_{1}}-X_{r_{2}}|)/E(|X'_{r_{1}}-X_{r_{2}}|^{2})=1-\sqrt{1-\rho }$ for the absolute value weights and $1-E(|X_{r_{1}}-X_{r_{2}}|^{2})/E(|X'_{r_{1}}-X_{r_{2}}|^{2})=\rho$ for the quadratic weights.

### Variance of *U*-Statistics

Let $U_{n}^{1}$ and $U_{n}^{2}$ be two *U*-statistics of *n* observations with symmetric kernels $\psi _{1}$, $\psi _{2}$ of dimension $k_{1}$ and $k_{2}$. Define

6.1 $$\begin{aligned} \sigma _{cc}^{2} = {{\,\textrm{Cov}\,}}(E[\psi _{1}(X_{1},\ldots ,X_{k_{1}})\mid X_{1},\ldots ,X_{c})],E[\psi _{2}(X_{1},\ldots ,X_{k_{2}})\mid X_{1},\ldots ,X_{c})]). \end{aligned}$$

#### Proposition 2

The exact covariance of $U_{1}^{n}$ and $U_{2}^{n}$ is

$$\begin{aligned} {{\,\textrm{Cov}\,}}(U_{1}^{n},U_{2}^{n})=\left( {\begin{array}{c}n\\ k_{1}\end{array}}\right) ^{-1}\sum _{c=1}^{k_{1}} \left( {\begin{array}{c}k_{2}\\ c\end{array}}\right) \left( {\begin{array}{c}n-k_{2}\\ k_{1}-c\end{array}}\right) \sigma _{cc}^{2}. \end{aligned}$$

If $k_{1}$ and $k_{2}$ are fixed, its asymptotic variance is $n{{\,\textrm{Cov}\,}}(U_{1}^{n},U_{2}^{n})\rightarrow k_{1}k_{2}\sigma _{12}$.

#### Proof

See (Lee, 2019, Theorem 2, p. 17) and (Lee, 2019, Theorem 2, p. 76). $\square$

### Expanding the Definitions

Here is sketch of how we could expand the definitions in Sect. 2 to encompass more complicated scenarios. We restrict ourselves to $g=2$, but the analysis can be expanded to arbitrary *g*. Suppose that any finite number of raters *R* is possible, the raters are not exchangeable, and that not every item is rated by every rater.

Let *X* denote a rating, *R* be the raters, and *I* be the items rated. Suppose we sample pairs $(X_{1},R_{1},I_{1}),(X_{2},R_{2},I_{2})$ independently from the same distribution *F*. Then we may define

6.2 $$\begin{aligned} D_{d}= & {} E[d(X_{1},X_{2})\mid I_{1}=I_{2},R_{1}\ne R_{2}],\nonumber \\ C_{d}= & {} E[d(X_{1},X_{2})\mid R_{1}\ne R_{2}],\nonumber \\ F_{d}= & {} E[d(X_{1},X_{2})]. \end{aligned}$$

These quantities have natural sample analogues; e.g.,

$$\begin{aligned} \hat{D}_{d}=N^{-1}\sum _{i=1}^{n}\sum _{r_{1}\ne r_{2}}d(x_{ir_{1}},x_{ir_{2}}), \end{aligned}$$

where *N* is the total number of paired observations and the rater indices run over the raters who observed at the *i*th observation *x*. Population and sample definitions of Cohen’s kappa and Fleiss’ kappa follow as laid out in the main text, e.g., $\kappa _{d}=1-D_{d}/C_{d}$.

**Table 8:** Coverage (first entry) and lengths (second entry) of confidence intervals: Perreault–Leigh model, Fleiss’s kappa.

	Method		Fleiss’s kappa: Perreault–Leigh model								
			$R=2$			$R=5$			$R=20$		
		*n*	10	40	100	10	40	100	10	40	100
*Weights*											
Nominal	Basic		0.82	0.95	0.95	0.93	0.95	0.95	0.96	0.95	0.96
			0.55	0.30	0.18	0.41	0.18	0.11	0.23	0.09	0.06
	Arcsine		0.98	0.94	0.94	0.98	0.95	0.95	0.95	0.95	0.95
			0.76	0.30	0.18	0.44	0.18	0.11	0.23	0.09	0.06
	Fisher		0.97	0.96	0.96	0.96	0.96	0.95	0.95	0.94	0.95
			0.95	0.32	0.19	0.51	0.19	0.11	0.24	0.09	0.06
Quadratic	Basic		0.65	0.86	0.92	0.85	0.93	0.94	0.94	0.95	0.95
			0.60	0.39	0.26	0.50	0.26	0.16	0.34	0.14	0.08
	Arcsine		0.83	0.89	0.93	0.89	0.94	0.94	0.94	0.95	0.95
			0.82	0.39	0.25	0.56	0.26	0.16	0.34	0.14	0.08
	Fisher		0.96	0.91	0.94	0.91	0.94	0.95	0.93	0.95	0.95
			0.98	0.44	0.27	0.67	0.27	0.16	0.37	0.14	0.08
Absolute value	Basic		0.81	0.92	0.94	0.91	0.95	0.95	0.95	0.95	0.95
			0.57	0.33	0.21	0.44	0.21	0.13	0.27	0.11	0.06
	Arcsine		0.99	0.93	0.95	0.94	0.95	0.95	0.95	0.95	0.95
			0.79	0.33	0.21	0.48	0.21	0.13	0.27	0.11	0.06
	Fisher		0.97	0.95	0.95	0.96	0.95	0.95	0.94	0.95	0.95
			0.97	0.36	0.21	0.56	0.21	0.13	0.28	0.11	0.07

**Table 9:** Coverage (first entry) and lengths (second entry) of confidence intervals: Normal model, Fleiss’s kappa.

	Method		Fleiss’s kappa: normal model								
			$R=2$			$R=5$			$R=20$		
		*n*	10	40	100	10	40	100	10	40	100
*Weights*											
Quadratic	Basic		0.89	0.93	0.95	0.90	0.95	0.94	0.90	0.93	0.95
			0.70	0.33	0.20	0.50	0.23	0.14	0.42	0.20	0.12
	Arcsine		0.90	0.93	0.95	0.90	0.95	0.94	0.90	0.92	0.94
			0.71	0.32	0.20	0.49	0.23	0.14	0.42	0.20	0.12
	Fisher		0.92	0.93	0.95	0.89	0.94	0.94	0.88	0.92	0.94
			0.74	0.33	0.20	0.51	0.23	0.14	0.43	0.20	0.12
Absolute value	Basic		0.92	0.95	0.95	0.91	0.96	0.93	0.90	0.93	0.93
			0.71	0.32	0.20	0.47	0.21	0.13	0.39	0.18	0.11
	Arcsine		0.92	0.95	0.95	0.90	0.95	0.92	0.89	0.93	0.93
			0.69	0.32	0.20	0.46	0.21	0.13	0.39	0.18	0.11
	Fisher		0.92	0.95	0.95	0.90	0.95	0.93	0.89	0.93	0.93
			0.68	0.32	0.20	0.46	0.21	0.13	0.39	0.18	0.11

**Table 10:** Coverage (first entry) and lengths (second entry) of confidence intervals: Perreault–Leigh model, Fleiss’ kappa ($R=5$).

	Method		Perreault–Leigh model, Fleiss’ kappa								
			$g=3$			$g=4$			$g=5$		
		*n*	10	40	100	10	40	100	10	40	100
*Weights*											
$V(d_{0})$	Arcsine		0.98	0.96	0.95	0.98	0.95	0.95	0.98	0.95	0.95
			0.41	0.16	0.1	0.4	0.16	0.1	0.38	0.15	0.09
	Fisher		0.96	0.96	0.95	0.96	0.96	0.95	0.97	0.96	0.95
			0.46	0.17	0.1	0.45	0.16	0.1	0.43	0.15	0.09
$V(d_{1})$	Arcsine		0.94	0.95	0.96	0.93	0.95	0.95	0.94	0.95	0.95
			0.5	0.21	0.13	0.46	0.19	0.11	0.46	0.19	0.11
	Fisher		0.95	0.95	0.95	0.96	0.96	0.96	0.95	0.95	0.95
			0.57	0.21	0.13	0.52	0.19	0.12	0.52	0.19	0.12
$V(d_{2}^{2})$	Arcsine		0.88	0.94	0.94	0.88	0.94	0.94	0.88	0.94	0.94
			0.59	0.26	0.16	0.59	0.26	0.16	0.59	0.26	0.16
	Fisher		0.91	0.94	0.95	0.91	0.94	0.95	0.9	0.94	0.95
			0.68	0.27	0.16	0.68	0.27	0.16	0.68	0.27	0.16
Hubert	Arcsine		0.98	0.96	0.96	0.97	0.96	0.96	0.97	0.96	0.96
			0.52	0.22	0.13	0.61	0.26	0.16	0.71	0.31	0.19
	Fisher		0.97	0.96	0.96	0.97	0.96	0.96	0.97	0.97	0.96
			0.58	0.22	0.13	0.67	0.27	0.16	0.77	0.31	0.19

#### Krippendorff’s Alpha

Now suppose that the ratings can take on only a finite number *C* distinct values. Define $o_{ck}$ as the number of times a pair of raters has classified an item into *c* and *k*, i.e.,

$$\begin{aligned} o_{ck}=\sum _{i=1}^{n}\sum _{r_{1}\ne r_{2}}1[x_{ir_{1}}=c,x_{ir_{2}}=k]. \end{aligned}$$

Then $N=\sum _{c,k}o_{ck}$ and $\hat{D}_{d}=N^{-1}\sum _{c,k}o_{ck}d(c,k).$ Moreover, define $n_{c}$ as the number of items classified as *c*. Then $n_{c}=\sum _{k}o_{ck}$, $\sum _{c}n_{c}=N$, and $\sum _{c,k}n_{c}n_{k}d(c,k)=N^{2}\hat{F}_{d}.$

##### Proposition 3

Using the above definitions, $\hat{\alpha }_{d}=\hat{\pi }_{d}+\frac{1}{N}(1-\hat{\pi }_{d})$. Since there are $N=2Rn$ rating pairs in the rectangular setup used in Sect. 2, $\hat{\alpha }_{d}=\hat{\pi }_{d}+\frac{1}{2Rn}(1-\hat{\pi }_{d})$ in that case.

##### Proof

The definition of $\hat{\alpha }_{d}$ can be found on Krippendorff (2018, p.235),

$$\begin{aligned} \hat{\alpha }_{d}=1-(N-1)\frac{\sum _{c\ne k}o_{ck}d(c,k)}{\sum _{c\ne k}n_{c}n_{k}d(c,k)}. \end{aligned}$$

From the above definitions, and the fact that $d(c,k)=0$ when $c=k$, we find that

$$\begin{aligned} \sum _{c\ne k}o_{ck}d(c,k)=\sum _{c,k}o_{ck}d(c,k)=N\hat{D}_{d}. \end{aligned}$$

In the same way,

$$\begin{aligned} \sum _{c\ne k}n_{c}n_{k}d(c,k)=\sum _{c,k}n_{c}n_{k}d(c,k)=N^{2}\hat{F}_{d}. \end{aligned}$$

Thus,

$$\begin{aligned} \hat{\alpha }_{d}=1-\frac{(N-1)}{N}\frac{\hat{D}_{d}}{\hat{F}_{d}} =1-\frac{\hat{D}_{d}}{\hat{F}_{d}}+\frac{1}{N}\frac{\hat{D}_{d}}{\hat{F}_{d}}, \end{aligned}$$

and using that $\hat{\pi }_{d}=1-\frac{\hat{D}_{d}}{\hat{F}_{d}}$, we are done. $\square$

### Proof of Correspondence with Gwet (2021)

Using the nominal disagreement function, Gwet (2021) uses the following estimator for the asymptotic variance of the pairwise Fleiss’ kappa:

$$\begin{aligned} \hat{\sigma }^{2}=\frac{1}{n-1}\sum _{i=1}^{n}(\kappa _{i}^{\star }-\hat{\kappa })^{2}. \end{aligned}$$

Translating into our notation (dropping the dependence on the disagreement *d*), we have that $\hat{\kappa }=1-\hat{D}/\hat{F}$. Moreover, one can verify that $\kappa _{i}^{\star }$ equals

$$\begin{aligned} \kappa _{i}^{\star }=1-\frac{\hat{\mu }(x_{i})}{\hat{F}} -2\frac{\hat{D}}{\hat{F}}\left( 1-\frac{\hat{\mu }_{F}(x_{i})}{\hat{F}}\right) , \end{aligned}$$

where $\hat{\mu }(x_i)$ and $\hat{\mu }_F(x_i)$ were defined in Sect. 4.

Following a small reorganization of the terms, we find that

$$\begin{aligned} \frac{1}{n-1}\sum _{i=1}^{n}(\kappa _{i}^{\star }-\hat{\kappa })^{2} =\frac{1}{\hat{F}^{2}}\frac{1}{n-1}\sum _{i=1}^{n}\left( 2\frac{\hat{D}}{\hat{F}}\left[ \hat{\mu }_{F}(x_{i})-\hat{F}\right] -[\hat{\mu }_{d}(x_{i}) -\hat{D}]\right) ^{2}. \end{aligned}$$

Using the definitions of $\hat{\sigma }_{D}^{2},\hat{\sigma }_{FD}$ and $\hat{\sigma }_{F}^{2}$ (c.f. Section 4.2), one can verify using simple algebraic manipulations that

$$\begin{aligned} \frac{1}{n-1}\sum _{i=1}^{n}\left( \kappa _{i}^{\star }-\hat{\kappa }\right) ^{2} = \frac{1}{\hat{F}^{2}}\left( \hat{\sigma }_{D}^{2}-2\hat{\sigma }_{FD} \frac{\hat{D}_{d}}{\hat{F}_{d}}+\hat{\sigma }_{F}^{2}\frac{\hat{D}_{d}^{2}}{\hat{F}_{d}^{2}}\right) ; \end{aligned}$$

hence, the estimator of Gwet (2021) is a special case of the proposed estimator in Sect. 4.2.

### Simulation of Fleiss’s Kappa

Here are the results of the simulation study in 5.1 for Fleiss’s kappa (Tables 8, 9, 10).

## Footnotes

1 For instance, Fleiss (1971), in his paper introducing Fleiss’ kappa, removed several ratings from this data to make sure the total number of ratings was 6 for each item.

2 Note that the concordance correlation coefficient is an intraclass correlation coefficient, see (Carrasco & Jover, 2003, p. 850).

3 The Schuster–Smith coefficient also encompasses the case of $2<g<R$ provided their weight function *v*(*s*) is appropriately defined, see the discussion on dispersion weights in (Schuster and Smith, 2005).

4 The Fréchet mean and variances are usually defined slightly differently, using $l^{2}(x,x_{k})$ instead of $l(x,x_{k})$, with *l* being a metric. Our definition of the Fréchet mean is sometimes called the generalized Fréchet mean or the $\alpha$-Fréchet mean.

5 We use fewer simulations (1, 000) than in the previous simulation (10, 000) since estimation is far more computationally expensive when dealing with continuous data, as it does not allow for binning.
